# Left Ventricular Diastolic Dysfunction in ARDS Patients

**DOI:** 10.3390/jcm11205998

**Published:** 2022-10-11

**Authors:** Paolo Formenti, Silvia Coppola, Laura Massironi, Giacomo Annibali, Francesco Mazza, Lisa Gilardi, Tommaso Pozzi, Davide Chiumello

**Affiliations:** 1 Department of Anesthesia and Intensive Care, ASST Santi Paolo e Carlo, San Paolo University Hospital, 20142 Milan, Italy; 2 Division of Cardiology, Department of Health Sciences, San Paolo Hospital, University of Milan, 20142 Milan, Italy; 3 Department of Health Sciences, University of Milan, 20142 Milan, Italy; 4 Coordinated Research Center on Respiratory Failure, University of Milan, 2014 Milan, Italy

**Keywords:** ARDS, left ventricular diastolic dysfunction, total lung weight

## Abstract

Background: The aim of this study was to evaluate the possible presence of diastolic dysfunction and its possible effects in terms of respiratory mechanics, gas exchange and lung recruitability in mechanically ventilated ARDS. Methods: Consecutive patients admitted in intensive care unit (ICU) with ARDS were enrolled. Echocardiographic evaluation was acquired at clinical PEEP level. Lung CT-scan was performed at 5 and 45 cmH_2_O. In the study, 2 levels of PEEP (5 and 15 cmH_2_O) were randomly applied. Results: A total of 30 patients were enrolled with a mean PaO_2_/FiO_2_ and a median PEEP of 137 ± 52 and 10 [9–10] cmH_2_O, respectively. Of those, 9 patients (30%) had a diastolic dysfunction of grade 1, 2 and 3 in 33%, 45% and 22%, respectively, without any difference in gas exchange and respiratory mechanics. The total lung weight was significantly higher in patients with diastolic dysfunction (1669 [1354–1909] versus 1554 [1146–1942] g) but the lung recruitability was similar between groups (33.3 [27.3–41.4] versus 30.6 [20.0–38.8] %). Left ventricular ejection fraction (57 [39–62] versus 60 [57–60]%) and TAPSE (20.0 [17.0–24.0] versus 24.0 [20.0–27.0] mL) were similar between the two groups. The response to changes of PEEP from 5 to 15 cmH_2_O in terms of oxygenation and respiratory mechanics was not affected by the presence of diastolic dysfunction. Conclusions: ARDS patients with left ventricular diastolic dysfunction presented a higher amount of lung edema and worse outcome.

## 1. Introduction

The most severe form of acute respiratory failure is ARDS, which is defined by inflammatory edema, increase in vascular permeability and the impairment of gas exchange [1,2]. Although the current ARDS definition excludes any form of hydrostatic pulmonary edema, the absence of invasive pulmonary monitoring can hide a possible cardiac failure (systolic, diastolic dysfunction) or fluid overload [3,4]. Diastolic function is affected by an active relaxation and passive compliance [5,6,7]. Thus, diastolic dysfunction is defined by the presence of any alteration in the relaxation, distensibility or filling of the left ventricle with a preserved systolic function [8,9]. The common predisposing factors are the presence of obesity, arterial hypertension, or diabetes mellitus, the female sex and ageing [10,11]. In addition, the possible presence of sepsis, hypoxemia, inflammation activation and cytokine release can promote a systolic/diastolic dysfunction [11,12]. The diastolic dysfunction in sepsis/septic shock ranged from 20% to 67% according to the definition applied and was reversible in patients who survived [11]. Moreover, in septic shock, the diastolic dysfunction was the strongest independent predictor of early mortality, better than cardiac biomarkers, even after adjusting for the severity of the disease, stroke volume and arterial hypoxemia [11,13,14]. In ARDS patients, the requirement of positive mechanical ventilation, fluid load and vasopressor in the early phase and during the weaning phase, leading to a possible increase in the venous return and afterload, can increase the risk of diastolic dysfunction and pulmonary edema [3,4]. Several studies including meta-analyses reported that patients who failed the weaning process presented a higher incidence of diastolic dysfunction [15,16,17,18,19]. Echocardiography has been recommended as the primary noninvasive clinical examination for evaluating the presence of diastolic dysfunction [20,21]. Several echocardiographic measurements for categorizing the diastolic dysfunction have been previously recommended; however, these measurements could not be applied in critically ill patients due to the presence of tachycardia or atrial fibrillation [21]. A more simplified definition based on Doppler echocardiography, which computes the ratio between the early diastolic velocity of mitral inflow to mitral annular velocity (E/e’), has been suggested [22]. This parameter showed a strong association with the left atrial pressure and with the categorization of patients with diastolic dysfunction according to the previous American Society of Echocardiography guidelines [22]. Although the diastolic dysfunction has been described since 1998 and accounted for more than 50% of the total heart failure, its role in ARDS patients is not well known. The aim of this study was to evaluate the possible presence of diastolic dysfunction by a simplified approach with echocardiography in the early phase of ARDS patients receiving mechanical ventilation. The secondary aim was to detect differences between patients with and without diastolic dysfunction in terms of respiratory mechanics, gas exchange and lung recruitability.

## 2. Materials and Methods

### 2.1. Study Design

This was a prospective observational study conducted between October 2017 and September 2019 at intensive care unit of San Paolo Hospital, Milan, Italy. The protocol was approved by the institutional review board and written consent was obtained according to the regulation applied (n. 7185/2019).

### 2.2. Patients

**Figure 1:**
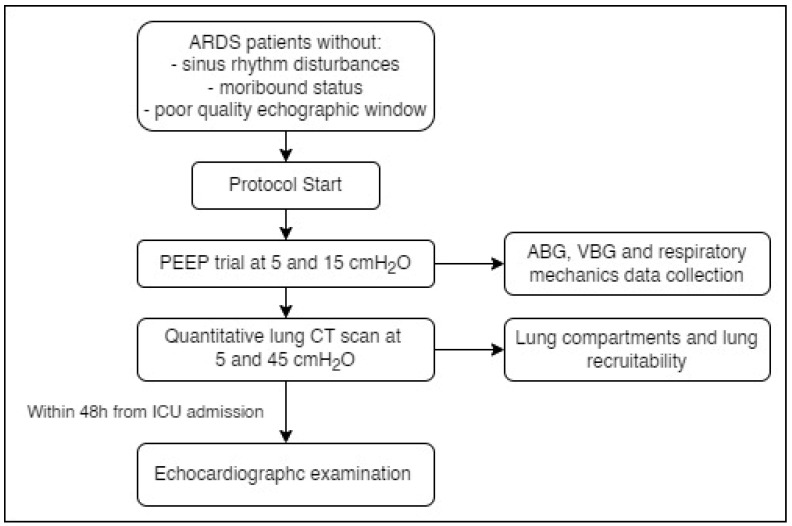
Study protocol flow chart. ARDS: Acute Respiratory Distress Syndrome; ABG: arterial blood gas; VBG: venous blood gas; CT: computed tomography; ICU: intensive care unit.

All consecutive patients admitted with ARDS according to the Berlin definition were enrolled. Exclusion criteria were absence of a sinus rhythm, a moribund status and a poor-quality echocardiographic images and measurements and a known history of diastolic dysfunction. At baseline patients were sedated, paralyzed and managed according to a lung protective ventilation strategy (tidal volume between 6–8 mL/Kg of ideal body weight) with a PEEP set to achieve an arterial saturation between 93–97%. Briefly, after a recruitment maneuver, a PEEP trial, (at two different levels of PEEP, 5 and 15 cmH_2_O randomly applied) by maintaining constant the tidal volume and oxygen fraction, was performed. At each PEEP level, blood gas analysis and partitioned respiratory mechanics measurements were performed. After the PEEP trial, patients underwent a lung CT scan performed in 2 different static conditions at 5 cmH_2_O of PEEP and at 45 cmH_2_O of airway plateau pressure [23] (Figure 1). A quantitative CT scan lung analysis using dedicated software (Maluna) was applied to compute the total lung gas, volume, weight and its amount of the different compartments (not inflated, poorly inflated, well inflated and overinflated tissue) [24]. The lung recruitability was computed as the difference between not-inflated lung tissue at 5 cmH_2_O and 45 cmH_2_O of airway plateau pressure to the total lung tissue at 5 cmH_2_O of PEEP [23].

### 2.3. Clinical Data

We collected demographic information, vital signs, mechanical ventilation parameters, CT scan quantitative analysis and echocardiographic measurements within 48 h from ICU admission.

### 2.4. Measurements

Esophageal pressure was measured using a standard balloon catheter (Smart Cath, Viasys, PalmSprings, CA, USA) consisting of a tube 103 cm long with an external diameter of 3 mm and a thin-walled balloon 10 cm long. The esophageal catheter was emptied of air and introduced trans orally into the esophagus to reach the stomach at a depth of 50–55 cm from the mouth. Subsequently the balloon was inflated with 1.5 mL of air. The intragastric position of the catheter was confirmed by a positive pressure deflection of intra-abdominal pressure during an external manual epigastric pressure. Subsequently, the catheter was retracted and positioned in the low esophageal position. The amount of gas in the balloon was periodically checked throughout the experiment. During an end-inspiratory and end-expiratory pause the airway and esophageal pressure were measured. The respiratory system, lung and chest wall elastance were computed according to the standard formulas [24]. To measure the functional residual capacity (FRC), a gas mixture of oxygen and 13.44% helium was manually equilibrated with the patient’s lung using a ventilation bag for 10 large insufflations [25]. Thereafter, helium concentration in the bag was analyzed (KG850, Hitech Instruments, Pennsburg, PA, USA). The FRC was computed as:

FRC (mL) = (Vb × Ci)/Cf − Vb

where Vb is the initial volume of the bag, Ci is the initial helium concentration and Cf is the final helium concentration after equilibration.

### 2.5. Transthoracic Echocardiography

Echocardiography was performed during controlled mechanical ventilation at 5 cmH_2_O of PEEP within 48 h of ICU admission. All echocardiographic examinations were performed and analyzed by a single trained cardiologist not involved in the patient care and blinded to the treatment and outcome (LM). Transthoracic echocardiography (TTE) was performed using a GE, Vivid E9 (General Electric Horten, Norway) equipped with a 2–5 MHz transducer. All images were obtained with standard techniques using M-mode, two-dimensional, and Doppler measurements in accordance with the ASE/EACVI recommendations [26]. Pulsed Doppler echocardiography was used to evaluate transmitral LV filling velocities at the tips of the mitral valve. The peak early-diastolic flow velocity (E) and the peak late-diastolic velocity (A) shown as the E/A ratio were measured by analyzing trans-mitral flow. The ASE/EACVI recommendations showed the four recommended variables for identifying left ventricular diastolic dysfunction (LVDD), and their abnormal cut-off values were annular e’ velocity (septal e’ < 7 cm/s or lateral e’ < 10 cm/s), average E/e’ ratio > 14, LA volume index > 34 mL/m^2^, and peak TR velocity > 2.8 m/s. Diastolic function was normal when more than half of the available variables did not meet the cut-off values for identifying abnormal function. LVDD was present when more than half of the available parameters met these cut-off values but was indeterminate when only half met these values. Patients were graded as the DD classification in three groups (I grade–II grade–III grade) according to the algorithm for the diagnosis of LVDD in ASE/EACVI recommendations. In addition to assessing diastolic function, we also measured the following parameters: the LV end-diastolic and end-systolic volumes, diameters and areas, the left ventricular ejection fraction (EF) using biplane modified Simpson’s rule, miocardical mass, thickness of septum and posterior wall, the RV end-diastolic diameter, the RV end-diastolic and end-systolic areas, the tricuspidal annular plane systolic excursion (TAPSE), RIMP (right index of myocardial performance) defined as the ratio of isovolumic time divided by Ejection Time (ET), or [(isovolumic relaxation time (IVRT) + isovolumic contraction time (IVCT))/ET].

### 2.6. Statistical Analysis

Continuous data are expressed as mean ± SD or median [IQR], as appropriate, while categorical data are expressed as %, number. Comparisons between patients with and without diastolic dysfunction for continuous data were performed by Student’s T test or the Wilcoxon–Mann–Whitney test. Comparisons between patients with and without diastolic dysfunction for categorical variables were performed by χ^2^ test. We investigated the role of diastolic dysfunction and PEEP on respiratory mechanics, gas exchange, hemodynamics and quantitative CT-scan data by two-ways repeated measures analysis of variance (ANOVA) between groups (diastolic dysfunction) and a fixed factor within-subjects PEEP. In case of statistically significant interaction, the comparisons of the two classes of a factor within each class of the other factor were performed with all pairwise multiple comparison procedures (Holm–Šidák method). The statistical analysis was performed using RStudio (R foundation for statistical computing, Vienna, Austria). A *p* value of 0.05 or less was considered statistically significant.

## 3. Results

A total of 30 patients were enrolled in this study. The baseline characteristics are shown in Table 1. The main sources of ARDS were pulmonary pneumonia (25 patients, 83%). The mean PaO_2_/FiO_2_ was 137 ± 52 with a median PEEP level of 10 [9–10] cmH_2_O; the applied median tidal volume of predicted body weight was 6.9 [6.6–7.3] mL/Kg PBW. The echocardiography study was performed within 48 h after the start of mechanical ventilation. 9 patients (30%) had a diastolic dysfunction with a grade 1, 2 and 3 in 33%, 45% and 22%, respectively. There was no difference in terms of PaO_2_/FiO_2_, applied tidal volume predicted body weight and minute ventilation between the two groups at the baseline; the FRC was not different (630 ± 230 vs. 600 ± 410 mL). The 28-day mortality was significantly worse in patients with diastolic dysfunction.

### 3.1. Echocardiographic Data

According to the echocardiographic criteria, patients with diastolic dysfunction had a significant higher E/e’ ratio and left atrial volume compared to patients without diastolic dysfunction 10.0 [8.9–14.7] versus 7.2 [6.1–9.2] and 65.0 [39.0–98.0] versus 37.0 [26–48] L/m^2^. The median ejection fraction 60 [56–61] %, none had an ejection fraction lower than 30% and was not different between two groups and similarly the end diastolic volume was not different between the two groups (Table 2). The TAPSE and the ratio between the RV end diastolic and the LV end diastolic areas were not different among the two groups (20.0 [17.0–24.0] versus 24.0 [20.0–27.0] mL; 0.66 [0.57–0.70] versus 0.66 [0.57–0.88] respectively) (Table 3).

### 3.2. Gas Exchange and Respiratory Mechanics Response to PEEP

The oxygenation was similar at 5 cmH_2_O of PEEP and similarly was the changes at 15 cmH_2_O of PEEP (Table 4 and Table S2). The carbon dioxide and the ratio between the ETCO_2_ and PaCO_2_ were not different. Concerning the total, lung and chest wall elastance were not different among the two groups both at 5 and 15 cmH_2_O of PEEP (Table 4).

### 3.3. Lung CT Scan Quantitative Data and Recruitability

Patients with diastolic dysfunction had a significantly higher total tissue weight compared to patients without diastolic dysfunction. The percentages of non aerated, poorly aerated, well aerated and overinflated tissue were similar between the two groups. The lung recruitability was similar in patients with diastolic dysfunction (33.3 [27.3–41.4] %) and in patients without diastolic dysfunction (30.6 [20.0–38.8] %) (See Table S1).

## 4. Discussion

The major findings of this this observational study, which applied a simplified echocardiographic examination in ARDS patients, were: (1) the LV diastolic dysfunction was present in 30% of the patients, and (2) the gas exchange and respiratory mechanics were not different between patients with and without LV diastolic dysfunction, while (3) the 28-day mortality and lung weight were significantly higher in patients with LV diastolic dysfunction. The most severe form of acute respiratory failure is ARDS, which is characterized by a severe deterioration in gas exchange due to an inflammatory pulmonary edema [1]. Pneumonia and sepsis were the most frequent causes of ARDS [27]. In the early phase, ARDS due to infection/inflammation is characterized by hypovolemia due to an increased capillary permeability, higher venous capacitance, vasoplegia and the requirement of mechanical ventilation. These factors associated with the patient’s history and comorbidities (age, hypertension, diabetes, renal failure) may promote hemodynamic failure, which has been reported in up of 60% of ARDS patients [3]. Thus, an early evaluation of heart failure of both LV ventricular and diastolic component is of paramount importance to improve the outcome. The diastole is a complex phase of the cardiac cycle that leads to ventricular filling before the ejection, and a normal diastolic phase is essential for ensuring an adequate preload and, consequently, cardiac output. The main determinants of the LV preload are the LV relaxation and compliance of the LV. The best assessment of LV relaxation (i.e., diastolic dysfunction) component is through an invasive measurement of the LV pressure by the pulmonary artery catheter [3,4]. However, based on some negative trials the pulmonary artery catheter, it is rarely used in critically ill patients. Thus, the assessment of possible presence of LV diastolic dysfunction remains challenging [4]. However, in critically ill patients, evidence has suggested performing an echocardiographic examination as soon as signs of hemodynamic failure are suspected [3,4], specifically evaluating the presence of possible diastolic dysfunction by a simplified echocardiographic examination based on the ratio of the early diastolic velocity of transmitral flow to the early myocardial relaxation wave (E/e’) [26]. The e’ is measured by tissue doppler imaging as the early diastolic mitral anulus velocity and the E reflects the early diastolic transmitral flow velocity, both dependent on LV relaxation. This ratio has been validated as an accurate predictor of pulmonary capillary wedge pressure in patients with cardiogenic shock and in the perioperative patients during cardiac surgery [28,29]. In this vein, Mousavi et al. showed in patients with septic shock a good correlation between the mean E/e’ ratio and pulmonary capillary wedge pressure [30]. Previous studies also showed that in septic shock the E/e’ ratio had an independent and better prognostic prediction of hospital outcome in septic shock compared to traditional cardiac biomarkers (BNP, NT, proBNP and TnT) [14]. According to previous data, in non-ARDS patients, diastolic dysfunction can be defined accordingly to echocardiographic examinations by an E/e’ ratio higher than 14 with a normal left ventricular ejection fraction [26].

In the present study, diastolic dysfunction was found in up to 30% patients, although only 22% of these presented a diastolic dysfunction of high grade. All patients had ARDS of different severity, and echocardiographic examinations were performed within 48 h from the start of mechanical ventilation. Patients with diastolic dysfunction were characterized by a worse 28-day outcome. Applying similar echocardiographic criteria, in severe sepsis/septic shock, LV diastolic dysfunction was found in 9% of the patients and was associated to a worse outcome [22,31]. This study also evaluated the possible associations between the diastolic dysfunction and gas exchange, partitioned respiratory mechanics (lung and chest wall elastance) and quantitative lung CT scan. The response to the change in PEEP in terms of oxygenation and respiratory mechanics from 5 to 15 cm H_2_O was not affected by the presence of diastolic dysfunction. On the contrary, the lung weight was significantly higher in patients with diastolic dysfunction. This suggested that the presence of diastolic dysfunction, (i.e., a higher filling pressure) could have increased the amount of lung fluid [16]. Similarly, the presence of diastolic dysfunction during the weaning phase associated with a reduction in the intrathoracic pressure and higher venous return promotes an increase in filling pressure. Previous studies and meta-analysis showed that diastolic dysfunction was associated with weaning failure due to the development of pulmonary edema promoted by an increase in filling pressure when the mechanical support was reduced [16,17,19]. However, it is not clear if the left ventricular diastolic dysfunction is mainly promoted by ARDS, pneumonia, sepsis, mechanical ventilation or fluid infusion or is rather only a preexisting condition in critically ill patients worsened by the underlying disease [32].

## 5. Conclusions

In conclusion, this study showed that LV diastolic dysfunction should be considered in ARDS patients because ARDS patents with left ventricular diastolic dysfunction presented a higher amount of lung edema and worse outcomes. Similar to patients with severe sepsis and septic shock, in whom diastolic dysfunction is one of the strongest independent predictors of mortality, ARDS could be associated with worse outcomes. In addition, the presence of diastolic dysfunction and fluid overload could be particularly harmful, promoting interstitial edema, tissue hypoxia and organ dysfunction [11,12,13,31]. However, further studies are needed to evaluate the diastolic function in the early phase of ARDS.

## Figures and Tables

**Table 1:** Characteristics of the study population within the first 48 h since ICU admission according to the presence or absence of diastolic dysfunction. BMI: body mass index; ICU: intensive care unit; ARDS: acute respiratory distress syndrome; PaO_2_: arterial oxygen partial pressure; FiO_2_: inspired oxygen fraction; PaCO_2_: arterial carbon dioxide partial pressure; PEEP: positive end-expiratory pressure; IBW: ideal body weight. Comparisons for continuous variables were performed by Student’s T test or Wilcoxon–Mann–Whitney test, as appropriate, while comparisons for categorical variables were performed by χ^2^ test.

	Study Population N = 30	Diastolic Dysfunction N = 9	No Diastolic Dysfunction N = 21	*p*
Age, years	63 ± 16	73 ± 11	60 ± 16	0.018
Male sex, % (number)	80 (24)	78 (7)	81 (17)	0.999
Weight, kg	72 ± 17	72 ± 12	72 ± 19	0.939
BMI, kg/m^2^	25 ± 5	26 ± 4	25 ± 6	0.077
History of, % (n)				
Hypertension	57 (17)	55 (5)	57 (12)	0.999
Myocardial infarction	23 (7)	33 (3)	19 (4)	0.706
Chronic kidney failure	17 (5)	11 (1)	19 (4)	0.952
Chronic obstructive pulmonary disease	17 (5)	22 (2)	14 (3)	0.968
Diabetes mellitus	37 (11)	44 (4)	33 (7)	0.869
SAPS II	43 ± 6	43 ± 5	44 ± 6	0.687
SOFA score	4 [2–5]	3 [2–5]	4 [2–5]	0.341
ICU stay, days	16 [12–22]	16 [9–16]	19 [12–27]	0.173
28 days mortality, % (n)	50 [15]	8 (89)	7 (33)	0.017
Diastolic dysfunction grade, % (n)				-
Grade I	33 (3)	33 (3)		
Grade II	45 (4)	45 (4)		
Grade III	22 (2)	22 (2)		
Cause of ARDS, % (number)				
Pulmonary	83 (25)	78 (7)	83 (18)	0.883
Extrapulmonary	17 (5)	22 /2)	17 (3)	0.579
PaO_2_/FiO_2_, mmHg	137 ± 52	115 ± 47	147 ± 52	0.119
PaCO_2_, mmHg	50 [41–59]	51 [45–70]	50 [41–56]	0.469
Arterial pH	7.39 ± 0.05	7.39 ± 0.04	7.39 ± 0.06	0.958
PEEP, cmH_2_O	10 [9–10]	10 [10–10]	10 [8–10]	0.818
Respiratory rate, breaths per minute	16 [15–18]	18 [17–20]	16 [15–17]	0.080
Respiratory system elastance, cmH_2_O/L	26 ± 7	22.5 ± 6.2	28.2 ± 7.6	0.118
Minute ventilation, L/min	7.6 ± 1.2	8.0 ± 1.4	7.4 ± 1.1	0.225
**Tidal volume per IBW, mL/kg IBW**	6.9 [6.6–7.3]	6.8 [6.6–7.3]	7.0 [6.5–7.3]	0.803
Functional residual capacity, mL	600 ± 360	630 ± 230	600 ± 410	0.770
Amine requirement, % (n)	66 (20)	78 (7)	62 (13)	0.564
Serum creatinine, mg/dL	1.1 [0.7–1.6]	1.0 [0.7–1.5]	1.2 [0.9–1.7]	0.482
Daily fluid balance, mL	200 [–300–800]	250 [–400–550]	250 [–250–350]	0.437

**Table 2:** Left ventricular and atrial echocardiographic characteristics according to the presence or absence of diastolic dysfunction. E: peak early-diastolic flow velocity; A: peak late-diastolic velocity; e‘: annular velocity. Comparisons for continuous variables were performed by Student’s T test or Wilcoxon–Mann–Whitney, as appropriate, while comparisons for categorical variables were performed by χ^2^ test.

	Study Population N = 30	Diastolic Dysfunction N = 9	No Diastolic Dysfunction N = 21	*p*
**Left Ventricle**				
End-diastolic diameter (mm)	44.5 [40.2–49-5]	50.0 [47.0–52.0]	42.0 [40.0–47.0]	0.027
End-systolic diameter (mm)	30.0 [25.2–36.0]	40.0 [36.0–42.0]	30.0 [25.0–32.0]	0.044
End-diastolic area (cm^2^)	17.0 [13.2–22.8]	20.0 [17.0–23.4]	15.0 [12.0–20.0]	0.442
End-diastolic volume (mL)	81.5 [68.5–108.0]	103.0 [72.0–130.0]	78.0 [66.0–103.0]	0.291
End-systolic volume (mL)	34.0 [25.0–51.5]	53.0 [35.0–67.0]	30.0 [23.0–44.0]	0.114
Ejection fraction (%)	60 [56–61]	57 [39–62]	60 [57–60]	0.396
Septum thickness (mm)	10.0 [8.0–11.0]	10.0 [10.0–13.0]	10.0 [8.0–10.0]	0.040
Posterior wall thickness (mm)	9.0 [8.0–10.0]	10.0 [8.0–10.0]	9.0 [8.0–10.0]	0.482
Myocardical mass (g/m^2^)	74.5 [58.5–97.2]	103.0 [91.0–111.0]	60.0 [45.0–87.0]	0.001
**Left Atrium**				
Left atrial volume index (mL/m^2^)	39.5 [32–60]	65.0 [39.0–98.0]	37.0 [26.0–48.0]	0.017
**Diastolic function**				
E (cm/s)	80.5 [64.0–93.8]	83.0 [67.0–92.0]	80.0 [64.0–95.0]	0.888
**A (cm/s)**	78.5 [56.0–92.5]	85.0 [54.0–93.0]	78.0 [59.0–91.0]	0.853
E/A ratio	1.1 [0.9–1.4]	0.9 [0.8–1.5]	1.2 [0.9–1.3]	0.910
e’ septal (cm/s)	8.0 [6.0–11.0]	6.1 [5.6–7.2]	9.3 [7.0–12.0]	0.085
e’ lateral (cm/s)	10.5 [7.8–13.4]	7.6 [6.4–8.6]	12.0 [10.0–14.2]	0.001
E/e’ ratio	8.6 [6.3–10.0]	10.0 [8.9–14.7]	7.2 [6.1–9.2]	0.013
Deceleration time (ms)	184 [162–253]	183 [170–253]	185 [160–253]	0.928

**Table 3:** Right ventricular and pulmonary artery echocardiographic characteristics according to the presence or absence of diastolic dysfunction. RV ED-area: right ventricular end-diastolic area; LV ED-area: left ventricular end-diastolic area; TAPSE: tricuspid annular plane systolic excursion; RIMP: right ventricular index of myocardial performance; PAPs: pulmonary arterial systolic pressure. Comparisons for continuous variables were performed by Student’s T test or Wilcoxon–Mann–Whitney, as appropriate, while comparisons for categorical variables were performed by χ^2^ test.

	Study Population N = 30	Diastolic Dysfunction N = 9	No Diastolic Dysfunction N = 21	*p*
**Right Ventricle**				
End-diastolic diameter (mm)	38.0 [33.5–39.8]	39.5 [29.5–42.0]	37.0 [34.5–38.3]	0.600
End-diastolic area (cm^2^)	17.0 [14.0–20.0]	17.0 [15.0–19.0]	17.0 [10.8–20.8]	0.906
RV ED-area/LV ED-area	0.67 [0.56–0.76]	0.66 [0.57–0.70]	0.66 [0.57–0.88]	0.647
End-systolic area (cm^2^)	9.0 [6.0–13.7]	6.5 [6.0–10.0]	10.0 [6.7–15.0]	0.465
Lateral wall (mm)	4.0 [3.0–4.0]	4.0 [4.0–4.0]	4.0 [3.0–4.5]	0.852
TAPSE (mm)	23.5 [19.0–26.5]	20.0 [17.0–24.0]	24.0 [20.0–27.0]	0.253
RIMP	0.30 [0.24–0.37]	0.31 [0.25–0.37]	0.30 [0.22–0.38]	0.662
Fractional shortening (%)	50 [43–57]	58 [47–60]	50 [41–50]	0.153
**Pulmonary Artery**				
Peak tricuspidal velocity (m/s)	2.5 [2.3–2.9]	2.8 [2.3–3.0]	2.4 [2.1–2.8]	0.404
PAPs (mmHg)	41 [31–46]	43 [31–49]	38 [31–44]	0.748

**Table 4:** Respiratory mechanics and gas exchange data according to the presence or absence of diastolic dysfunction. ERS: respiratory system elastance; EL: lung elastance; ECW: chest wall elastance; PaO_2_: arterial oxygen partial pressure; FiO_2_: inspired oxygen fraction; PaCO_2_: arterial carbon dioxide partial pressure; EtCO_2_: end-tidal carbon dioxide partial pressure. The role of the of PEEP between groups was assessed by two-way repeated-measures analysis of variance (ANOVA) followed by all-pairwise comparisons (Holm–Sidak method).

	Study Population N = 30	Diastolic Dysfunction N = 9	No Diastolic Dysfunction N = 21	p disf	p PEEP	p inter
End Inspiratory Airway Pressure (cmH_2_O)						
5 cmH_2_O	16.4 [13.7–18.5]	15.9 [13.4–17.6]	16.4 [13.8–18.5]	0.420	<0.001	0.725
15 cmH_2_O	24.7 [22.1–26.7]	24.0 [22.0–25.4]	25.2 [23.8–26.8]			
Driving Pressure (cmH_2_O)						
5 cmH_2_O	10.1 [7.7–12.9]	8.3 [6.9–9.7]	11.4 [7.8–13.5]	0.226	0.435	0.152
15 cmH_2_O	9.5 [8.0–11.9]	9.0 [7.6–11.4]	10.1 [8.6–12.0]			
E_RS_ (cmH_2_O/L)						
5 cmH_2_O	20.9 [16.7–28.1]	18.7 [16.9–20.5]	26.8 [16.7–29.4]	0.658	0.187	0.211
15 cmH_2_O	21.4 [18.2–28.5]	20.2 [20.0–28.1]	21.4 [17.0–28.7]			
E_L_ (cmH_2_O/L)						
5 cmH_2_O	16.2 [12.0–25.0]	14.7 [12.8–24.0]	19.0 [11.2–25.2]	0.555	0.140	0.588
15 cmH_2_O	15.2 [12.0–21.0]	15.8 [14.9–21.4]	14.0 [11.2–19.9]			
E_CW_ (cmH_2_O/L)						
5 cmH_2_O	4.8 [3.0–6.3]	4.0 [3.1–5.8]	5.5 [2.9–6.3]	0.585	0.288	0.544
15 cmH_2_O	5.9 [3.9–10.3]	4.6 [3.8–8.1]	6.3 [5.0–10.5]			
PaO_2_ (mmHg)						
5 cmH_2_O	70 [63–76]	71 [70–74]	68 [62–79]	0.842	<0.001	0.821
15 cmH_2_O	88 [75–128]	107 [97–134]	87 [68–100]			
PaO_2_/FiO_2_						
5 cmH_2_O	128 [107–146]	117 [84–146]	131 [110–145]	0.274	<0.001	0.745
15 cmH_2_O	187 [113–237]	195 [94–242]	182 [131–232]			
PaCO_2_ (mmHg)						
5 cmH_2_O	47.4 [40.4–56.4]	45.9 [39.4–51.0]	50.2 [41.5–58.0]	0.836	0.808	0.023
15 cmH_2_O	48.7 [43.2–52.4]	48.9 [45.3–52.0]	47.2 [43.0–52.5]			
EtCO_2_/PaCO_2_						
5 cmH_2_O	0.74 [0.69–0.82]	0.70 [0.68–0.74]	0.76 [0.70–0.83]	0.073	0.905	0.229
15 cmH_2_O	0.75 [0.68–0.84]	0.69 [0.66–0.76]	0.77 [0.73–0.89]			
Ventilatory Ratio						
5 cmH_2_O	1.4 [1.3–1.7]	1.3 [1.2–1.9]	1.5 [1.2–1.8]	0.111	0.089	0.231
15 cmH_2_O	1.6 [1.5–1.9]	1.7 [1.2–1.8]	1.6 [1.3–1.8]			

## Data Availability

Not applicable.

## Supplementary Materials

The following supporting information can be downloaded at: https://www.mdpi.com/article/10.3390/jcm11205998/s1, Table S1. Quantitative computed-tomography scan data according to the presence or absence of diastolic dysfunction, Table S2. Hemodynamic characteristics according to the presence or absence of diastolic dysfunction.
