# Supplementary material for: Left Ventricular Diastolic Dysfunction in ARDS Patients
Source: J Clin Med. 2022 Oct 11;11(20):5998. doi: 10.3390/jcm11205998 (PMC9604741; doi:10.3390/jcm11205998)
Supplement: Supplementary file 1 [file jcm-11-05998-s001.zip › jcm-1910394-supplementary.pdf]

Table S1. Quantitative Computed-Tomography scan data according to the presence or absence of diastolic dysfunction. LPR: lung potential recruitment.

The role of the of PEEP between groups was assessed by two-ways repeated measures Analysis Of Variance (ANOVA) followed by all-pairwise comparisons (Holm-Sidak method).

	**Study population N = 30**	**Diastolic dysfunction N = 9**	**No diastolic dysfunction N=21**	p_{group}	p_{PEEP}	$p_{interaction}$
Total lung weight (g)						
5 cmH_2O	1440 [1169 – 1934]	1669 [1354 – 1909]	1554 [1146 – 1942]	**0.004**	**0.002**	**0.010**
15 cmH_2O	1630 [1207 – 1957]	1626 [1176 – 1711]	1634 [1299 – 1962]			
Total lung gas volume (mL)						
5 cmH_2O	808 [455 – 1381]	977 [712 – 1409]	613 [426 – 1295]	0.722	**<0.001**	0.338
15 cmH_2O	2356 [1353 – 3183]	2206 [1523 – 2920]	2500 [1297 – 3248]			
Not aerated tissue (g)						
5 cmH_2O	631 [515 – 1152]	631 [536 – 1043]	631 [510 – 1315]	0.651	**<0.001**	0.762
15 cmH_2O	253 [107 – 640]	217 [139 – 432]	409 [99 – 641]			
Not aerated tissue (%)						
5 cmH_2O	50 [40 – 59]	51 [45 – 56]	49 [37 – 62]	0.542	**<0.001**	0.366
15 cmH_2O	29 [14 – 45]	13 [11 – 36]	30 [21 – 44]			
Poorly aerated tissue (g)						
5 cmH_2O	505 [301 – 566]	566 [342 – 669]	420 [269 – 535]	0.482	0.497	0.173
15 cmH_2O	427 [239 – 655]	424 [273 – 628]	430 [232 – 664]			
Poorly aerated tissue (%)						
5 cmH_2O	28 [21 – 33]	32 [30 – 42]	25 [21 – 32]	0.097	**0.004**	**0.002**
15 cmH_2O	27 [19 – 33]	29 [22 – 39]	26 [19 – 33]			
Well aerated tissue (g)						
5 cmH_2O	276 [130 – 432]	245 [209 – 422]	307 [130 – 436]	0.887	**<0.001**	0.856
15 cmH_2O	662 [459 – 853]	655 [617 – 767]	668 [448 – 858]			
Well aerated tissue (%)						
5 cmH_2O	22 [11 – 32]	12 [11 – 26]	31 [11 – 34]	0.689	**<0.001**	0.210
15 cmH_2O	40 [29 – 53]	41 [32 – 51]	39 [26 – 52]			
Over inflated tissue (g)						
5 cmH_2O	0 [0 – 2]	0 [0 – 2]	0 [0 – 2]	0.548	**<0.001**	0.616
15 cmH_2O	8 [1 – 65]	9 [5 – 40]	7 [1 – 67]			
Over inflated tissue (%)						
5 cmH_2O	0 [0 – 0]	0 [0 – 0]	0 [0 – 1]	0.399	**0.048**	0.732
15 cmH_2O	0 [0 – 4]	0 [0 – 2]	0 [0 – 5]			
LPR (%)	32.5 [10.5 – 39.2]	33.3 [27.3 – 41.4]	30.6 [20.0 – 38.8]	0.456		

Table S2. Haemodynamic characteristics according to the presence or absence of diastolic dysfunction.

The role of the of PEEP between groups was assessed by two-ways repeated measures Analysis Of Variance (ANOVA) followed by all-pairwise comparisons (Holm-Sidak method).

	**Study population N = 30**	**Diastolic dysfunction N = 9**	**No diastolic dysfunction N=21**	p_{group}	p_{PEEP}	$p_{interaction}$
Heart rate (bpm)						
5 cmH_2O	89 [72 – 107]	70 [60 – 97]	94 [82 – 110]	0.059	0.532	0.607
15 cmH_2O	86 [65 – 104]	65 [58 – 100]	93 [82 – 111]			
SBP (mmHg)						
5 cmH_2O	126 [114 – 141]	127 [120 – 140]	125 [113 – 141]	0.773	**0.003**	0.349
15 cmH_2O	118 [106 – 132]	111 [109 – 133]	120 [106 – 128]			
DBP (mmHg)						
5 cmH_2O	60 [56 – 64]	60 [56 – 61]	58 [54 – 68]	0.636	0.338	0.340
15 cmH_2O	57 [53 – 66]	57 [55 – 64]	57 [52 – 67]			
MAP (mmHg)						
5 cmH_2O	81 [74 – 91]	88 [75 – 91]	80 [72 – 87]	0.733	**0.012**	0.585
15 cmH_2O	75 [71 – 86]	75 [73 – 90]	75 [69 – 85]			
CVP (mmHg)						
PEEP 5 cmH_2O	11 [9 – 13]	12 [8 – 13]	11 [10 – 13]	0.298	**<0.001**	0.342
PEEP 15 cmH_2O	14 [11 – 17]	14 [9 – 15]	14 [12 – 17]			
